# CART model to classify the drought status of diverse tomato genotypes by VPD, air temperature, and leaf–air temperature difference

**DOI:** 10.1038/s41598-023-27798-8

**Published:** 2023-01-12

**Authors:** Shih-Lun Fang, Yuan-Kai Tu, Le Kang, Han-Wei Chen, Ting-Jung Chang, Min-Hwi Yao, Bo-Jein Kuo

**Affiliations:** 1 Department of Agronomy, National Chung Hsing University, Taichung, 40227 Taiwan, ROC; 2 Division of Biotechnology, Taiwan Agricultural Research Institute, Taichung, 41362 Taiwan, ROC; 3 Division of Crop Science, Taiwan Agricultural Research Institute, Taichung, 41362 Taiwan, ROC; 4 Department of Agronomy, National Taiwan University, Taipei, 10617 Taiwan, ROC; 5 Division of Agricultural Engineering, Taiwan Agricultural Research Institute, Taichung, 41362 Taiwan, ROC; 6 Smart Sustainable New Agriculture Research Center (SMARTer), Taichung, 40227 Taiwan, ROC; 7 Innovation and Development Center of Sustainable Agriculture (IDCSA), National Chung Hsing University, Taichung, 40227 Taiwan, ROC

**Keywords:** Drought, Statistical methods

## Abstract

Regular water management is crucial for the cultivation of tomato (*Solanum lycopersicum* L.). Inadequate irrigation leads to water stress and a reduction in tomato yield and quality. Therefore, it is important to develop an efficient classification method of the drought status of tomato for the timely application of irrigation. In this study, a simple classification and regression tree (CART) model that includes air temperature, vapor pressure deficit, and leaf–air temperature difference was established to classify the drought status of three tomato genotypes (i.e., cherry type ‘Tainan ASVEG No. 19’, large fruits breeding line ‘108290’, and wild accession ‘LA2093’). The results indicate that the proposed CART model exhibited a higher predictive sensitivity, specificity, geometric mean, and accuracy performance compared to the logistic model. In addition, the CART model was applicable not only to three tomato genotypes but across vegetative and reproductive stages. Furthermore, while the drought status was divided into low, medium, and high, the CART model provided a higher predictive performance than that of the logistic model. The results suggest that the drought status of tomato can be accurately classified by the proposed CART model. These results will provide a useful tool of the regular water management for tomato cultivation.

## Introduction

Tomato (*Solanum lycopersicum* L.) is a popular vegetable worldwide. To bridge the seasonal gap of production and prevent the rainfall and unfavorable temperature from reducing the yield and quality, most tomatoes are cultivated in greenhouses to stabilize and mitigate these adverse environmental impacts^1^. However, water management remains the main issue for farmers even when cultivated under greenhouse conditions^2,3^.

The shortage of water resources is a major limiting factor for agricultural production in many regions. Additionally, the quality and yield of tomato are not only affected by genotype, but also related to water management. To improve crop quality and water use efficiency, a water stress is induced by applying a deficit irrigation or increasing the salinity of nutrient solution during cultivation^4,5^. Unfortunately, the water stress induced by underirrigation at the vegetative and reproductive periods of tomato leads to abnormal growth, aborted flowering, and fruit setting failure, which cause a significant reduction in yield and quality^6,7^. Under moderate water deficit, the photosynthesis is limited, but it can recover in a short time after irrigation. Conversely, if water shortage continues, the irrigation cannot reverse the photosynthesis^8^. Therefore, it is very important to apply the water stress at an optimum level. Different genotypes or growth stages may have various responses to water stress^9,10^. Most crops are drought sensitive at various growth stages. The flowering and fruit setting stages are most sensitive to water deficits in drip irrigated tomatoes^11^. To provide the breeding material to resist drought stress, various species of tomato have been studied^12–14^. Wild tomato is the most resilient against abiotic and biotic stress compared to the domesticated tomato^12,13^. Tapia et al.^14^ found better morpho-physiological responses such as tolerance to drought stress in wild tomato than those in domesticated tomato.

In order to achieve adequate water management, it is important to decide a suitable irrigation strategy, which relies on an accurate, reliable, and timely classification of the drought status of tomato^15–17^. The drought status of plants is mainly determined by the soil water content, morphological alternation, physiological responses, and gene expression^7,18–20^. Changing the soil moisture monitored through sensors has been criticized because of the spatial heterogeneity of soils can make the measurements unrepresentative^21–24^. Gene expression profiling cannot reflect the instant drought status in the greenhouse, while other kinds of stress may contribute to the same expression variation. In addition, these methods are time-consuming and labor-demanding, which limits the number of plants and scale of the experiment^19,25^. In contrast, the evaluation of the drought status by examining variations in physiological responses such as stomata conductance, transpiration rate, and leaf temperature by means of some instruments is relatively efficient and effortless^8,26–28^.

When a plant is under drought stress, the changes in stomatal closure are more sensitive and rapid than the water potential and leaf water content^29^. The stomatal closure is one of the major factors limiting plant photosynthesis under mild or moderate water stress^30^. Medrano et al.^8^ found that light-saturated stomatal conductance (g_sw_) can be used as a reference parameter to reflect the degree of drought for C_3_ plants. Besides, the species-effect of g_sw_ on photosynthesis seems to be lower than that of the leaf water potential and relative water content^8^. However, although g_sw_ provides information about the water status of plants, current methods of g_sw_ measurement are designed to be in physical contact with leaves, which is suitable to manually measure individual plant but not favorable for large-scale and field-scale scenarios. Other common indicators to depict the water status of plants are leaf temperature and leaf-to-air vapor pressure deficit (VPD). While plants suffer from drought stress, the stomatal closure reduces the heat emission and air efflux from leaves, leading to an increase in leaf temperature and VPD. Therefore, plant temperature and leaf–air temperature difference (T_diff_) can be used to indirectly assess the plant g_sw_^31–34^. The reported indicators for evaluating plant drought status by T_diff_ are stress degree day (SDD)^35^ and crop water stress index (CWSI)^36^. However, temperature-based indicators are strongly influenced by the VPD and air temperature (T_air_)^37–39^. Therefore, in the subsequent establishment of the drought status model, except for the T_diff_, both T_air_ and VPD will also be considered in this study as independent variables.

Logistic regression is a statistical method that can establish the relationship between predictive variables and binary (dichotomous) and/or ordinal dependent variables^40^. Logistic regression has been used in the analysis of plant disease risk factors, and implemented as disease predictive models to classify with or without disease of wheat, oilseed rape, pyrethrum, and peanut plants^40,41^. Other than logistic regression, classification and regression tree (CART) is a non-parametric regression procedure developed by Breiman et al.^42^ in 1984. CART supports a non-linear classification and is capable of handling collinearity between predictive variables^43^. Due to its flexibility, interpretability, and broad applicability, CART has been widely used as a classification algorithm for multiclass issues in agriculture, environmental protection, biomedicine, and computer science^44–47^.

For automated irrigation management in greenhouses, most farmers have used a timer to regularly drive irrigation or to maintain a specific soil water content. However, this method neglects to consider the plant response^15^. Sometimes, soil moisture may not accurately represent the plant water status, and different genotypes have various drought tolerance responses. If a traditional irrigation system is adopted, the problems of irrigation deficiencies or excesses often become unavoidable. Therefore, conducting the water management on the basis of plant response is more appropriate and accurate^18^. In our previous studies, plant temperature was utilized to classify the drought status of greenhouse tomato to improve irrigation system^15,48^. However, these studies did not consider the inferences of different genotypes and growth stages.

To facilitate and conduct proper water management, the goal of this study is to develop a simple discriminant model to instantly decide the drought status of tomatoes to serve as a rule for irrigation decision-making based on plant responses. The seedlings of cherry type tomato ‘Tainan ASVEG No. 19’ were subjected to drought and regular irrigation treatments, and the net CO_2_ assimilation rate (A_n_), g_sw_, VPD, T_air_, and T_diff_ parameters were collected each day after treatment. The drought status was divided into binary (WW: well-watered; WS: water deficit stress) and ordinal (L: low stress; M: medium stress; H: high stress) variables according to the value of g_sw_. The T_air_, VPD, and T_diff_ were used as explanatory variables to build the CART and logistic regression models for predicting the drought status of the tomato. Except for the data collected from ‘Tainan ASVEG No. 19,’ data of the wild accession ‘LA2093’ (*Lycopersicon pimpinellifolium*) and the large fruit breeding line ‘108290’ were used to evaluate the model applicability for different genotypes and growth stages of the tomato.

## Results and discussion

### Relationship between g_sw_ and A_n_

**Figure 1:**
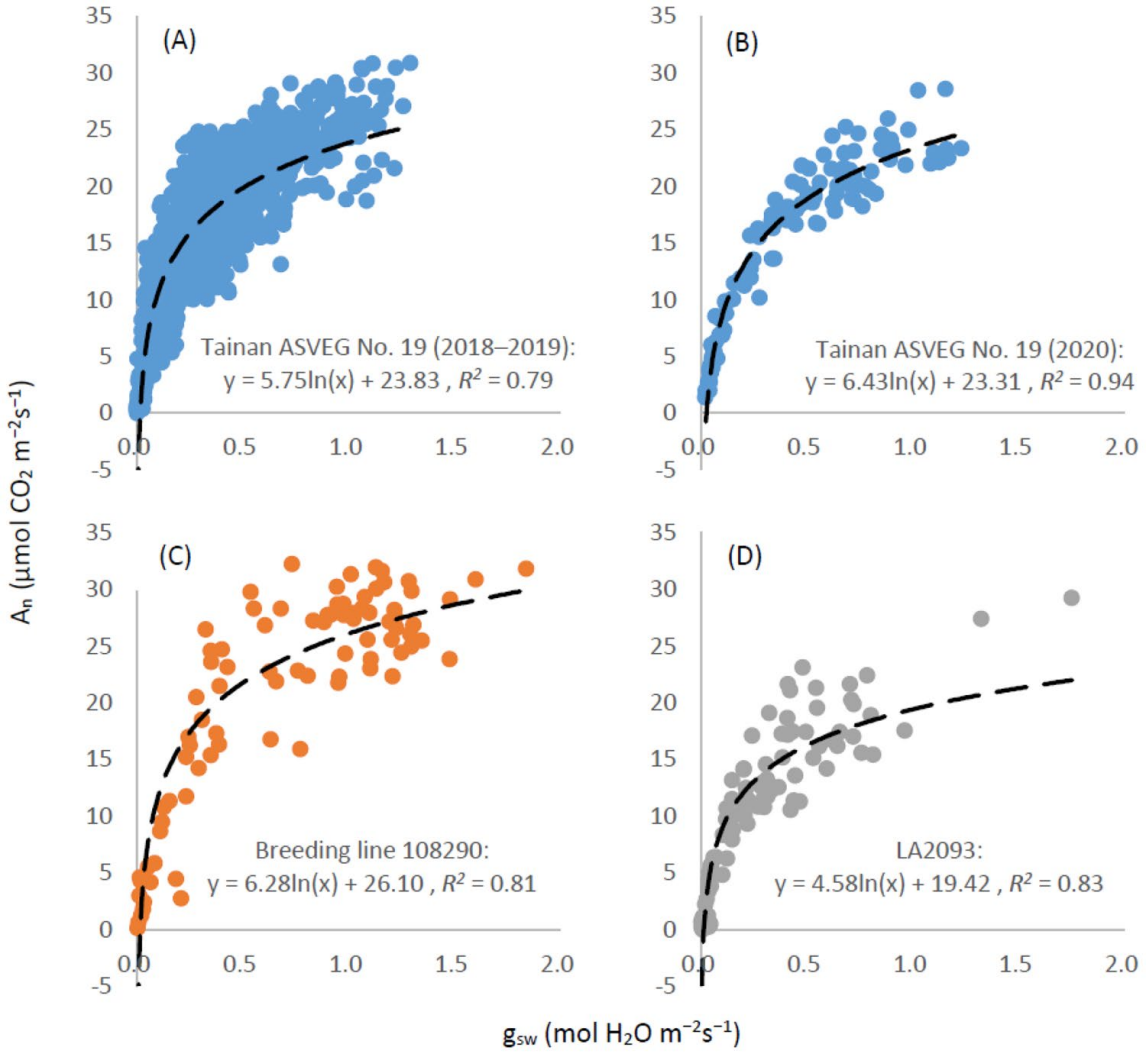
Relationship between light-saturated stomatal conductance (g_sw_) and net CO_2_ assimilation rate (A_n_) of (**A**) Tainan ASVEG No. 19 (2018–2019), (**B**) Tainan ASVEG No. 19 (2020), (**C**) breeding line 108290, and (**D**) LA2093.

The relationship between g_sw_ and A_n_ was displayed using a logarithmic function, and the coefficients of determination (*R*^2^) were 0.79–0.94 (Fig. 1). Thus, approximately 80% variation of A_n_ can be explained by g_sw_. In addition, a strong correlation was observed between A_n_ and g_sw_, irrespective of the data collected from different growth stages and genotypes; the Spearman correlation coefficients (*ρ*) were all above 0.77 (Table 1). In fact, under drought stress, plants close stomata to avoid excessive water loss. Therefore, the closure of stomata results in a lack of CO_2_ required for photosynthesis. On the other hand, the lack of water causes the dehydration of tissues that conduct photosynthesis and eventually impedes the photosynthesis efficiency of plants^49^. A high degree of correlation between g_sw_ and A_n_ was observed in field- and pot-grown plants^8,50^. These results strengthen our subsequent establishment of the drought status model based on g_sw_.

**Table 1:** Spearman correlation coefficient between light saturation g_sw_ and the parameters A_n_, VPD, T_air_, and T_diff_ in different datasets.

Dataset	A_n_	VPD	T_diff_	T_air_
Tainan ASVEG No. 19 (2018–2019)	0.87	− 0.78	− 0.76	0.26
Tainan ASVEG No. 19 (2020)	0.94	− 0.82	− 0.86	0.07
Breeding line 108290	0.78	− 0.77	− 0.68	0.05
LA2093	0.92	− 0.77	− 0.89	0.07

g_sw_: stomatal conductance; A_n_: net CO_2_ assimilation rate; VPD: vapor pressure deficit; T_diff_: leaf–air temperature difference; T_air_: air temperature.

### Relationship of g_sw_ with VPD, T_diff_, and T_air_

**Figure 2:**
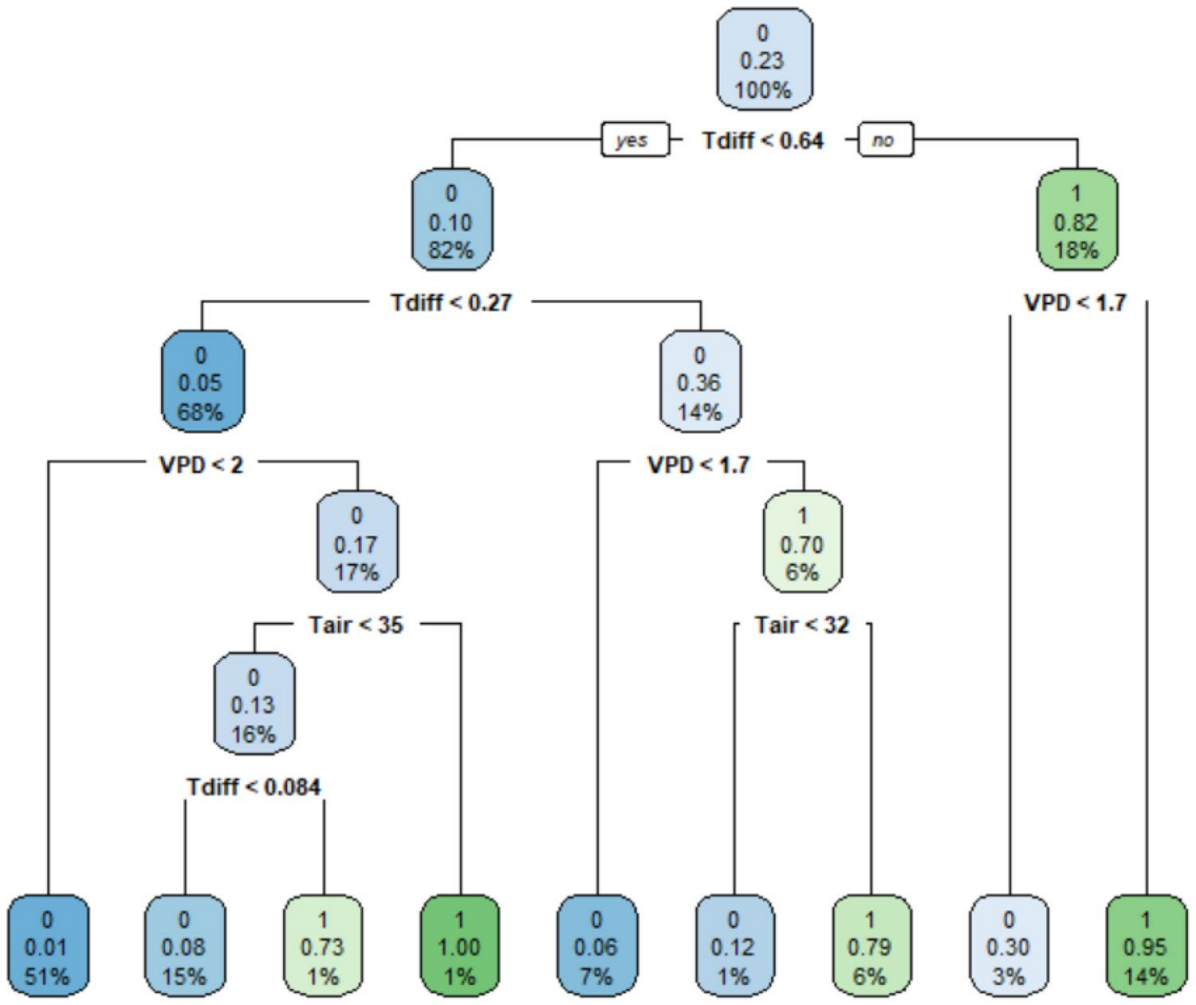
Classification and regression tree model for classifying binary drought status. At each intermediate node, a case goes to the left child node only if the condition is satisfied. Each node in the model has three values, the top value is the predicted class at this node, the second value is the probability that the Y = 1 (water deficit stress), and the third value is the number of observations for this node as a percentage of the whole dataset. The predicted class is a (0,1) variable, where 0 represents well-watered and 1 represents water deficit stress, respectively.

**Figure 3:**
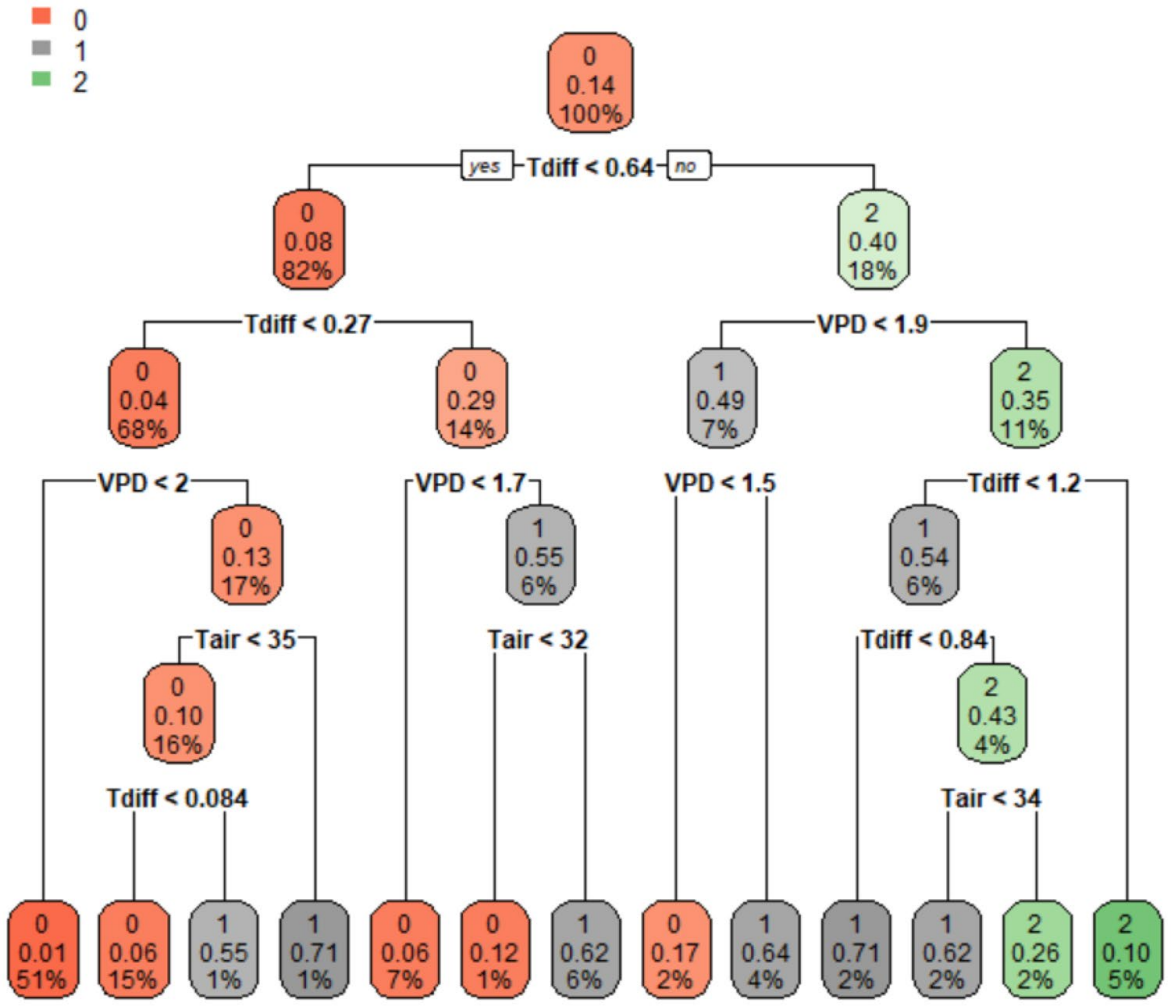
Classification and regression tree model for classifying ordinal drought status. At each intermediate node, a case goes to the left child node only if the condition is satisfied. Each node in the model has three values, the top value is the predicted class at this node, the second value is the probability that the Y = 1 (medium stress), and the third value is the number of observations for this node as a percentage of the whole dataset. The predicted class 0, 1, and 2 represent low, medium, and high stress, respectively.

VPD is one of the factors that induces stomatal changes in many plants^51^. Stomata close as the leaf-to-air VPD increases regardless of soil water conditions^8,51^. In the study, the *ρ* between g_sw_ and VPD for all genotypes was -0.77 to -0.82 (Table 1). In addition to reducing the efficiency of photosynthesis, stomatal closure also hinders heat loss through leaf transpiration, thereby increasing the plant temperature^27,52^. Therefore, T_diff_ should be negatively correlated with g_sw_. The result of this study indicated that the *ρ* between g_sw_ and T_diff_ for all genotypes was -0.68 to -0.89 (Table 1). Although the g_sw_ had a slight tendency to increase as T_air_ rose, the correlation between g_sw_ and T_air_ was very weak (*ρ* = 0.05–0.26) (Table 1). Raschke^53^ summarized the stomata feedback mechanism in which changes in temperature affect CO_2_ assimilation, and the open or closure of stomata that respond to temperature changes are influenced by the CO_2_ feedback. Urban et al.^54^ found that g_sw_ increases with rising T_air_. However, VPD is more important than T_air_ in the change in g_sw_^55^, highlighting the weak correlation between g_sw_ and T_air_. Even if T_air_, T_diff_, and VPD were put into the CART model or logistic model together, T_air_ is kept in the final model (Table 2; Figs. 2, 3). Thus, T_air_ may have some influence on the prediction of g_sw_.

**Table 2:** Logistic model parameters.

Model	Parameter	Estimate	Standard error
Binary	Intercept	− 25.10***	5.30
	VPD	2.77***	0.30
	T_air_	0.53**	0.16
	T_diff_	3.65***	0.30
Ordinal	Intercept 1	17.82***	3.67
	Intercept 2	21.22***	3.74
	VPD	− 2.51***	0.22
	T_air_	− 0.32**	0.11
	T_diff_	− 3.74***	0.24

Asterisks indicate the parameter significantly different from 0. **p < 0.01, ***p < 0.001.

### Classification ability of binary response models

In the study, 70% of the data of the Tainan ASVEG No. 19 (2018–2019) dataset were used to build the model. Next, the Tainan ASVEG No. 19 (2020), breeding line 108290, LA2093, and the remaining 30% data of Tainan ASVEG No. 19 (2018–2019) dataset were used as the testing sets for model validation.

The parameters of the logistic model in the model-building stage are shown in Table 2. When using the 30% data of Tainan ASVEG No. 19 (2018–2019) as the testing dataset, the classified performance of the logistic model revealed a sensitivity of 0.82, specificity of 0.96, geometric mean of 0.89, and 93.10% accuracy (Table 3). For the other testing datasets, the logistic model also had an acceptable performance with a sensitivity of 0.80–1.00, specificity of 0.79–0.85, geometric mean of 0.86–0.92, and 80.23–89.74% accuracy (Table 3).

**Table 3:** Performance of model validation for binary logistic model. Values that meet acceptable standards are shown in bold.

Testing dataset	Sensitivity	Specificity	Geometric mean	Accuracy
Tainan ASVEG No. 19 (2018–2019, 30%)	0.82	**0.96**	**0.89**	**93.10%**
Tainan ASVEG No. 19 (2020)	**1.00**	**0.85**	**0.92**	89.74%
Breeding line 108290	0.80	0.80	**0.80**	80.23%
LA2093	**0.93**	0.79	**0.86**	84.69%

The structure of the binary CART model is illustrated in Fig. 2. For the 30% data of Tainan ASVEG No. 19 (2018–2019) used as validation, the classified performance of the CART model displayed a sensitivity of 0.75, specificity of 0.97, geometric mean of 0.85, and accuracy of 92.18% (Table 4). The CART model revealed a comparable and better performance than that of the logistic model when using different testing datasets, with a sensitivity of 0.87–1.00, specificity of 0.89–0.93, geometric mean of 0.90–0.94, and 90.82–92.11% accuracy (Table 4).

**Table 4:** Performance of model validation for the classification and regression tree model (binary response). Values that meet acceptable standards are shown in bold.

Testing dataset	Sensitivity	Specificity	Geometric mean	Accuracy
Tainan ASVEG No. 19 (2018–2019, 30%)	0.75	**0.97**	**0.85**	**92.18%**
Tainan ASVEG No. 19 (2020)	**1.00**	**0.89**	**0.94**	**92.11%**
Breeding line 108290	**0.87**	**0.93**	**0.90**	**91.46%**
LA2093	**0.90**	**0.91**	**0.90**	**90.82%**

### Classification ability of ordinal response models

In the ordinal response models, the training and testing datasets were same as the binary response models. The performances of the classified ability of the ordinal logistic model are shown in Table 5. When using 30% of the data of Tainan ASVEG No. 19 (2018–2019) as the testing dataset, the correctly classified percentages of L, M, and H statuses were 98.98%, 59.02%, and 54.00%, respectively, and the overall accuracy of the classified performances was 87.38%. For the Tainan ASVEG No. 19 (2020) testing dataset, the correctly classified percentages of L, M, and H statuses were 81.48%, 23.81%, and 100.00%, respectively, and the overall accuracy was 72.81%. The performances of classifying the drought status for the breeding line 108290 and LA2093 datasets were between Tainan ASVEG No. 19 (2018–2019) and Tainan ASVEG No. 19 (2020). The values of the overall accuracy were 77.55% and 83.72%, respectively, for predicting the different drought status under different genotypes and growth stages (Table 5).

**Table 5:** Confusion matrix for the ordinal logistic model. Values that meet acceptable standards are shown in bold.

Actual class	Model predicted class			Correctly classified % per class	Overall accuracy
	L	M	H		
**Tainan ASVEG No. 19 (2018–2019, 30%)**					**87.38%**
L	290	3	0	98.98%	
M	20	36	5	59.02%	
H	2	21	27	54.00%	
**Tainan ASVEG No. 19 (2020)**					72.81%
L	66	12	3	81.48%	
M	9	5	7	23.81%	
H	0	0	12	100.00%	
**Breeding line 108290**					**83.72%**
L	65	3	3	91.55%	
M	1	3	2	50.00%	
H	5	0	4	44.44%	
**LA2093**					77.55%
L	51	5	1	89.47%	
M	5	7	1	53.85%	
H	2	8	18	64.29%	

L: low stress, with g_sw_ ≧ 0.15 mol H_2_O m^−2^ s^−1^; M: medium stress, with 0.05 ≦ g_sw_ < 0.15 mol H_2_O m^−2^ s^−1^; H: high stress, with g_sw_ < 0.05 mol H_2_O m^−2^ s^−1^.

The results and the structure of the multi-class CART model are represented in Table 6 and Fig. 3. When the CART model predicts the 30% testing dataset of the Tainan ASVEG No. 19 (2018–2019), the correctly classified percentages of L, M, and H statuses were 96.59%, 63.93%, and 58.00%, respectively, and the overall accuracy of the classification was 86.88%. For the Tainan ASVEG No. 19 (2020) testing dataset, the correctly classified percentages of L, M, and H statuses were 87.65%, 71.43%, and 75.00%, respectively, and the overall accuracy was 83.33%. For the breeding line 108290 and LA2093 datasets, the overall accuracy were 79.59 and 84.88%, respectively, for classifying the drought status (Table 6).

**Table 6:** Confusion matrix for the classification and regression tree model (multi-class). Values that meet acceptable standards are shown in bold.

Actual class	Model predicted class			Correctly classified % per class	Overall accuracy
	L	M	H		
**Tainan ASVEG No. 19 (2018–2019, 30%)**					**86.88%**
L	283	10	0	96.59%	
M	16	39	6	63.93%	
H	6	15	29	58.00%	
**Tainan ASVEG No. 19 (2020)**					**83.33%**
L	71	10	0	87.65%	
M	0	15	6	71.43%	
H	0	3	9	75.00%	
**Breeding line 108290**					**84.88%**
L	66	5	0	92.96%	
M	0	3	3	50.00%	
H	2	3	4	44.44%	
**LA2093**					79.59%
L	52	5	0	91.23%	
M	4	7	2	53.85%	
H	0	9	19	67.86%	

L: low stress, with g_sw_ ≧ 0.15 mol H_2_O m^−2^ s^−1^; M: medium stress, with 0.05 ≦ g_sw_ < 0.15 mol H_2_O m^−2^ s^−1^; H: high stress, with g_sw_ < 0.05 mol H_2_O m^−2^ s^−1^.

### Models comparison and evaluation

The binary response models performed well for the four datasets (Tables 3, 4). It is worth noting that the data used to build the model were taken from ‘Tainan ASVEG No. 19’ at the seedling/vegetative growth stage, while our testing data included ‘Tainan ASVEG No. 19’, breeding line ‘108290’, and ‘LA2093’ at the flowering stage. The acceptable performance of the binary response models indicate that the logistic and CART model have the potential to classify the binary drought status of tomatoes across different genotypes and growth stages.

As for the multi-class models, both logistic regression and CART revealed good classified ability for the L class, but poor performance in classifying M and H status (Tables 5, 6). The reason may be to the class-imbalanced datasets used in this study, as the number of cases of M and H categories were much lower than those of the L class (Table 7). Class imbalance may lead to poor recognition of M and H categories by the models and contribute to the declined classification capability^56,57^. The performance of the multi-class model can be further improved if the class number of the dataset was redistributed using some resampling methods^58,59^.

**Table 7:** Descriptions of the four datasets used in this study.

Dataset	Growth stage	Binary response		Ordinal response			Total (*n*)
		WW	WS	L	M	H	
Tainan ASVEG No. 19 (2018–2019)	Seedling/vegetative growth	957	281	957	176	105	1238
Tainan ASVEG No. 19 (2020)	Flowering	81	33	81	21	12	114
Breeding line 108290	Flowering	71	15	71	6	9	86
LA2093	Flowering	57	41	57	13	28	98

WW: well-watered, with g_sw_ ≥ 0.15 mol H_2_O m^−2^ s^−1^; WS: water deficit stress, with g_sw_ < 0.15 mol H_2_O m^−2^ s^−1^; L: low stress, with g_sw_ ≧ 0.15 mol H_2_O m^−2^ s^−1^; M: medium stress, with 0.05 ≦ g_sw_ < 0.15 mol H_2_O m^−2^ s^−1^; H: high stress, with g_sw_ < 0.05 mol H_2_O m^−2^ s^−1^.

When comparing the performances of the logistic and CART models, it can be found that the latter generally outperformed the former (Tables 3, 4, 5, 6). In the case of highly class-imbalanced data, unsatisfactory model performance for the logistic model was often observed^60^. Even if the logistic model has a good performance, it is difficult to interpret and visualize the classification process, contrary to the process of CART analysis^60^. In addition, the CART model makes fewer assumptions than those of the logistic regression and can deal with complex interactions and nonlinearities^61^. These properties contribute to the capability of the CART model to handle class-imbalance datasets^42,60,62^, outperforming the logistic model^60,63^. After comprehensively considering the classified performance and convenience of the application, the CART models were recommend for predicting the drought status of tomatoes. In application, only the air temperature, relative humidity, and plant temperature sensors need to be installed to achieve the values of input variables required by the model and set the decision rules of CART in the control system. Taking the decision rule on the rightmost of Fig. 2 as an example, when the T_diff_ $>$ 0.64 °C and the VPD $>$ 1.7 kPa, the tomato has a high probability (0.95) of being in a state of water shortage, indicating that it should be irrigated at this time.

## Conclusions

The proposed CART model with T_air_, VPD, and T_diff_ as independent variables had a good performance on predicting tomato drought status. The performance of the CART model was generally better than that of the logistic model both in binary and ordinal responses. In addition, the results indicated that the CART model can classify the WW and WS as well as the L, M, and H statuses for domesticated and wild tomato genotypes at different growth stages. Taking the advantages of the convenient measuring of input variables, good classified performances, and the intuitive visualization, the proposed CART model can be utilized as a simple and practical method to classify the drought status of diverse tomato genotypes at vegetative and reproductive stages. In fact, the proposed method only needs to install air temperature, relative humidity, and plant temperature sensors and sets the decision rules of CART in the greenhouse to control the water supply system. In the future, the data of water shortage in the fruiting stage can be taken into consideration to further verify the reliability of the model. The performance of the proposed model can be further improved if the class imbalance problem is solved.

## Methods

### Experimental layout

In order to develop a drought stress detection method across different growth stages (vegetative and reproductive stages) and genotypes, two experiments were conducted in the 1# and 2# solar greenhouses at the Taiwan Agricultural Research Institute (TARI) located in Taichung City, Taiwan (latitude 24° 01′ N, longitude 120° 41′ E). In the 1# greenhouse, the cherry tomato cultivar ‘Tainan ASVEG No. 19’ was used between 2018 and 2019. Eight young seedlings with 6–8 fully expanded leaves were planted in baskets (50 cm × 40 cm × 30 cm) with 6D soil substrate (BVB, De Lier, The Netherlands). The experiment contained two irrigation treatments, a regular watering and drought treatments. In the regular watering treatment, tomato was irrigated daily until the field capacity was reached. For the drought treatment, no irrigation was applied after transplanting to mimic a progressive drought condition. The substrate volumetric water content (SVWC) was determined by WaterScout SM100 (Spectrum Technologies, Aurora, IL, USA). Four digital sensors were inserted evenly into the substrate at 10 cm depth of each plastic basket. The SVWC was recorded every 30 min after the regular irrigation and drought treatments were applied to tomato seedlings. In total, the experiment in 1# greenhouse was performed seven times at different time points.

In the 2# greenhouse, except for ‘Tainan ASVEG No. 19’, wild accession ‘LA2093’ and large fruits breeding line ‘108290’ were planted in the peat moss during the 2020 summer. Tomatoes were planted at a density of approximately 27,900 plants/ha. Differing from the 1# greenhouse, the irrigation treatments (regular watering and drought treatments) started from the flowering stage to the fruit setting stage in the 2# greenhouse, because this period was most sensitive to water deficits in drip irrigated tomatoes^11^. The study complies with relevant institutional, national, and international guidelines and legislation.

### Environmental parameters and physiological data collection

For each tomato plant, 3–5 fully expanded leaves from the top of the plant were continuously measured. The leaf temperature, T_air_, A_n_, g_sw_, transpiration rate (E), and leaf-to-air VPD were measured using a LI-6800 portable photosynthesis system (LICOR Biosciences, Lincoln, NE, USA) at ambient air temperature (28.0–32.0 °C), air humidity (RH = 60%), reference CO_2_ concentration (400 μmol mol^−1^), and stable light intensity of 1200 μmol photons m^−2^ s^−1^ using an internal LED light source (red:blue = 9:1). Measurements were taken between 10:00 and 14:00. Data collection started from the drought treatment applied to the tomato showed clear signs of water shortage (Fig. S1), which was judged visually. The clear symptoms of water shortage were appeared about 2 to 3 weeks after drought treatment, when SVWC was 7–12%. In the study, the observations of ‘Tainan ASVEG No. 19’ in 2018–2019, ‘Tainan ASVEG No. 19’ in 2020, breeding line ‘108290’, and ‘LA2093’ are 1238, 114, 86, and 98, respectively.

### Relating the light-saturated stomatal conductance to environmental and physiological parameters

In this study, the relationship between the light-saturated g_sw_ of the tomatoes and A_n_ was first established. Next, the parameters VPD, T_air_, and T_diff_, which can affect or reflect the stomatal closure, were related to the light-saturated g_sw_. When building the relationship between light saturation g_sw_ and A_n_, several models i.e., linear regression, logarithmic curve, exponential curve, and polynomial regression were considered to find the best model using Excel 2016 software. Additionally, the Spearman correlation coefficients between light saturation g_sw_ and the parameters A_n_, VPD, T_air_, and T_diff_ were calculated.

### Data labeling

The tomato drought status was assessed using the thresholds for g_sw_ defined by Medrano et al.^8^. The thresholds were defined as follows: for binary response, WW, with g_sw_ ≥ 0.15 mol H_2_O m^−2^ s^−1^ and WS, with g_sw_ < 0.15 mol H_2_O m^−2^ s^−1^. For the ordinal response, L, with g_sw_ ≧ 0.15 mol H_2_O m^−2^ s^−1^; M, with 0.05 ≦ g_sw_ < 0.15 mol H_2_O m^−2^ s^−1^; and H, with g_sw_ < 0.05 mol H_2_O m^−2^ s^−1^. The whole data were divided into four datasets according to the data sources: (1) Tainan ASVEG No. 19 (2018–2019), (2) Tainan ASVEG No. 19 (2020), (3) breeding line 108290, and (4) LA2093. The description of the four datasets is provided in Table 7. After labeling the drought statuses, the differences of physiological parameter values between different drought statuses were examined. Because the assumption of normality was found to be violated by the Shapiro–Wilk test, the nonparametric methods were used for the comparison of different drought statuses. Mann–Whitney U and Kruskal–Wallis tests were used to examine the differences of E, A, and T_diff_ values between drought statuses for binary and ordinal responses, respectively.

For the binary response, all physiological parameter values of three genotypes differed between WW and WS statuses. The values of E and A_n_ of WW plants were significantly higher than those of WS plants. Conversely, the values of T_diff_ of WW plants were significantly lower than those of WS plants (Table S1). For the ordinal response, the values of E and A_n_ decreased with the increasing drought levels, and values of T_diff_ increased with the increasing drought levels. However, it was observed that the physiological parameter values of M and H statuses showed no significantly different, except LA2093 (Table S2).

### Models building and validation

Logistic regression is a modeling approach that can be used to describe the relationship between predictor variables and a dichotomous or multi-category response variable. For the tomato drought status defined in the previous section, a logistic model for *p*-1 independent variables was defined as follows:

1 $${\text{logit}}\left[ {{\text{P}}\left( {{\text{Y }} = { 1}} \right)} \right] \, = {\text{ ln}} \left[ {\frac{{{\text{P}}\left( {{\text{Y }} = { 1}} \right)}}{{1 - {\text{P}}\left( {{\text{Y }} = { 1}} \right)}}} \right] = a + b_{{1}} {\text{X}}_{{1}} + b_{{2}} {\text{X}}_{{2}} + \, \cdot\cdot\cdot \, + b_{{\text{k}}} {\text{X}}_{{{\text{p}} - {1}}}$$

where P(Y = 1) is the probability of WS status, given the values of X_1_,···, X_p−1_; *a* is an intercept; and *b*_1_, … , *b*_p−1_ are regression coefficients. Additionally, the probability of P(Y = 1) is 1/[1 + exp ($-$ *a* $-$ *b*_1_X_1_ $-$ *b*_2_X_2_ $-$ ··· $-$ *b*_p−1_X_p−1_)] in the multiple logistic regression model. It appears that the logistic model can be expressed as a logit form and is simplified as a linear function. For the final model, the threshold probability, i.e., the probability value to classify an observation to the WS status with the most accurate prediction result, was used as a classification criterion^41^.

For the *k*-class ordinal response data, one of the underlying assumptions for the ordinal logistic regression is that the regression coefficient of each independent variable is identical for each of the *k* $- 1$ cumulative logits, but different intercepts (Eq. 2)^41^.

2 $$\begin{aligned} & {\text{logit}}[{\text{P}}({\text{Y}} \le j)] \, = {\text{ ln}} \left[ {\frac{{{\text{P}}({\text{Y}} \le j)}}{{1 - {\text{P}}({\text{Y}} \le j)}}} \right] = a_{{\text{j}}} + b_{{1}} {\text{X}}_{{1}} + b_{{2}} {\text{X}}_{{2}} + \, \cdot\cdot\cdot \, + b_{{\text{h}}} {\text{X}}_{{{\text{p}} - {1}}} ,j = { 1},{ 2}, \, \ldots \, ,k-1; \\ & {\text{P}}({\text{Y}} \le k) \, = { 1 }\;{\text{and}}\;{\text{ P}}({\text{Y}} \le 0) \, = \, 0. \\ \end{aligned}$$

The probability function of predicting each category of drought statuses (L, M, and H) can be defined as per Eq. (3). The probability function given the highest probability value was the predicted drought status^41^.

3 $${\text{P}}\left( {{\text{Y }} = j} \right) \, = {\text{ P}}({\text{Y}} \le j) - {\text{P}}({\text{Y}} \le j - 1) \, ,\quad j = { 1},{ 2},{ 3}$$

The CART model is a common categorical classifier, which takes either continuous or categorical variable as predictor variables to predict the continuous dependent variable, requires no assumptions, and is simple to interpret^43^. It employs the recursive partitioning method using all predictor variables to split subsets of the dataset to create two child nodes, repeatedly^62^. Starting with the entire dataset, i.e., root node, the CART approach explores all possible values of the predictor variables to find the best predictor variable that can split the node. The best partition is one that minimizes the average impurity of the two child nodes. In this study, the Gini index of diversity was used to choose the best predictor at each node. The Gini index at node *t*, *g*(*t*) is expressed as follows:

4 $$g\left( t \right) \, = \mathop \sum \limits_{i \ne j} p\left( {j|t} \right)p\left( {i|t} \right)$$

where *i* and *j* are the different categories of the dependent variable.

Regardless of the method used to build the classification model, it was randomly selected 70% data of the Tainan ASVEG No. 19 (2018–2019) dataset as the training set, and used T_air_, VPD, and T_diff_ as independent variables to build the model. The remaining 30% data of the Tainan ASVEG No. 19 (2018–2019) dataset were used as the testing set for model validation. In addition, the Tainan ASVEG No. 19 (2020), breeding line 108290, and LA2093 datasets were used to validate the applicability of the models to different growth substrates, genotypes, and growth stages of the tomatoes. Since the stress responses vary under different conditions^64^, this validation can clarify the generalizability of the proposed model^65^.

### Discriminant ability of the models

For the binary class model, sensitivity, specificity, geometric mean, and accuracy were used to evaluate the model performance. The definition of these metrics is expressed as Eqs. (5)–(8), respectively:

5 $${\text{Sensitivity }} = \frac{{{\text{TP}}}}{{{\text{TP}} + {\text{FN}}}}$$

6 $${\text{Specificity }} = \frac{{{\text{TN}}}}{{{\text{TN}} + {\text{FP}}}}$$

7 $${\text{Geometric mean }} = \sqrt {{\text{Sensitivity}} \times {\text{Specificity}}}$$

8 $${\text{Accuracy }} = \frac{{{\text{TP}} + {\text{TN}}}}{{{\text{TP}} + {\text{TN}} + {\text{FP}} + {\text{FN}}}}$$

where TN is true negative (when the true drought status of the tomato was “WW,” and the model also classified it as “WW”); FP is false positive (when the true drought status of the tomato was “WW,” but the model classified it as “WS”); FN is false negative (when the true drought status of the tomato was “WS,” but the model classified it as “WW”); and TP is true positive (when the true drought status of the tomato was “WS,” and the model also classified it as “WS”).

The performance of the multi-class model were evaluated with the correctly classified percentage for each class and overall accuracy. Let us assume that a size *N* dataset includes *k* classes and each class has *n*_*i*_ instances (*i* = 1, 2, …, *k*), and *c*_ij_ are the elements of the *k* × *k* confusion matrix, where *i*, *j* = 1, 2, … , *k*. The rows and columns of the matrix show the true and predicted values at each class, respectively. Next, the correctly classified percentage for each class and overall accuracy can be defined using Eqs. (9)–(10):

9 $${\text{Correctly}}\;{\text{classified}}\;{\text{percentage}}\;{\text{for}}\;{\text{class}}\;i = \frac{{c_{ii} }}{{n_{i} }} ,\quad i = { 1},{ 2}, \, \ldots \, ,k$$

10 $${\text{Overall accuracy }} = \frac{{\mathop \sum \nolimits_{i = 1}^{k} c_{ii} }}{{\mathop \sum \nolimits_{i = 1}^{k} n_{i} }} = \frac{{\mathop \sum \nolimits_{i = 1}^{k} c_{ii} }}{N}$$

The range of the metrics described here is from 0 to 1. The closer the values of these metrics are to 1, the better the classification ability of the model. Model performance is considered acceptable if the sensitivity > 0.85, specificity > 0.85, geometric mean > 0.75, and accuracy > 90.00% for a binary response. The acceptable standard for the ordinal response model is that the overall accuracy is > 80.00%. These criteria are set based on the median (or close to the median) of previous water status classification studies^7,15,19,48,66,67^.

### Statistical analysis

The statistical analyses were implemented using the R software (version 4.1.3). Spearman correlation coefficients were calculated using the *cor* function. Binary logistic models were constructed using the *glm* function. The ordinal logistic model was built using the *vglm* function in *VGAM* package (version 1.1–7). The CART model was implemented using the *rpart* function in the *rpart* package (version 4.1.16).

## Supplementary Information

Supplementary Information.

## Data Availability

Data generated or analyzed during this study were included in this published article.
