# Supplementary material for: CART model to classify the drought status of diverse tomato genotypes by VPD, air temperature, and leaf–air temperature difference
Source: Sci Rep. 2023 Jan 12;13:602. doi: 10.1038/s41598-023-27798-8 (PMC9837056; doi:10.1038/s41598-023-27798-8)
Supplement: Supplementary file 1 — Supplementary Information. [file 41598_2023_27798_MOESM1_ESM.docx]

**Supplementary material**


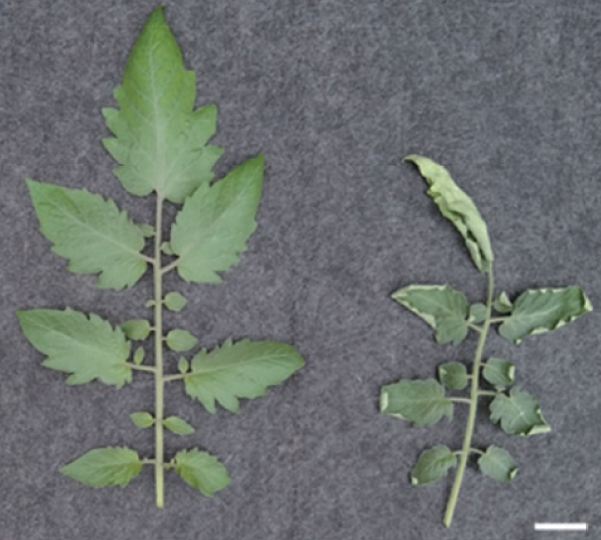


**Figure S1.**　The phenotypic changes of tomato leaves in the regular watering treatment (left) and drought treatment (right) (bar = 5 cm).

**Table S1.** The Mann-Whitney U test results of transpiration rate (E), net CO_2_ assimilation rate (A_n_), and leaf-air temperature difference (T_diff_). The values in the table are median, and the values in parentheses are interquartile range. Asterisks indicate significant differences between different drought statuses within the same genotype.

| Genotype | Status | E  (mmol H_2_O m^−2^s^−1^) | A_n_  (μmol CO_2_ m^−2^s^−1^) | T_diff_  (℃) |
| --- | --- | --- | --- | --- |
| Tainan ASVEG No. 19 | WW | 7.62^***^  (3.94) | 19.72^***^  (5.34) | −0.66^***^  (0.97) |
|  | WS | 1.57  (0.59) | 4.96  (3.14) | 0.88  (0.52) |
| Breeding line 108290 | WW | 7.97^***^  (4.46) | 25.56^***^  (6.20) | −0.75^***^  (0.96) |
|  | WS | 0.84  (1.49) | 4.29  (3.58) | 0.73  (0.87) |
| LA2093 | WW | 4.82^***^  (2.89) | 15.14^***^  (5.85) | −0.12^***^  (0.66) |
|  | WS | 0.86  (0.87) | 2.65  (5.12) | 1.15  (0.63) |

WW: well-watered, with g_sw_ ≥ 0.15 mol H_2_O m^−2^s^−1^; WS: water deficit stress, with g_sw_ < 0.15 mol H_2_O m^−2^s^−1^. ^*^*p*<0.05, ^**^*p*<0.01, ^***^*p*<0.001.

**Table S2.** The Kruskal-Wallis test results of transpiration rate (E), net CO_2_ assimilation rate (A_n_), and leaf-air temperature difference (T_diff_). The values in the table are median, and the values in parentheses are interquartile range. Different letters indicate significant differences between different drought statuses within the same genotype by Dunn post hoc test (*p*<0.05).

| Genotype | Status | E  (mmol H_2_O m^−2^s^−1^) | A_n_  (μmol CO_2_ m^−2^s^−1^) | T_diff_  (℃) |
| --- | --- | --- | --- | --- |
| Tainan ASVEG No. 19 | L | 7.62 ^a^  (3.94) | 19.72 ^a^  (5.34) | −0.66 ^b^  (0.97) |
|  | M | 1.87 ^b^  (0.90) | 5.99 ^b^  (3.16) | 0.74 ^a^  (0.46) |
|  | H | 1.24 ^b^  (0.25) | 3.22 ^b^  (1.19) | 1.26 ^a^  (0.48) |
| Breeding line 108290 | L | 7.97 ^a^  (4.46) | 25.56 ^a^  (6.20) | −0.75 ^b^  (0.96) |
|  | M | 2.26 ^b^  (0.86) | 7.24 ^b^  (3.67) | 0.82 ^a^  (0.30) |
|  | H | 0.36 ^b^  (0.29) | 2.38 ^b^  (3.06) | 0.40 ^a^  (1.49) |
| LA2093 | L | 4.82 ^a^  (2.89) | 15.14 ^a^  (5.85) | −0.12 ^c^  (0.66) |
|  | M | 1.91 ^b^  (0.91) | 6.37 ^b^  (4.01) | 0.74 ^b^  (0.50) |
|  | H | 0.50 ^c^  (0.72) | 0.70 ^c^  (2.45) | 1.33 ^a^  (0.53) |

L: low stress, with g_sw_ ≧ 0.15 mol H_2_O m^−2^s^−1^; M: medium stress, with 0.05 ≦ g_sw_ < 0.15 mol H_2_O m^−2^s^−1^; H: high stress, with g_sw_ < 0.05 mol H_2_O m^−2^s^−1^
